# Suppression of Glucagon-Like Peptide-1 Release by Inhibition of Intestinal NLRP3 Inflammasome Activation in Asc^–/–^ and Nlrp3^–/–^ Mice

**DOI:** 10.3389/fphys.2019.01213

**Published:** 2019-10-01

**Authors:** Yu Chen, Jason Kidd, Owais M. Bhat, Xinxu Yuan, Jinni Hong, Xingxiang He, Pin-Lan Li

**Affiliations:** ^1^Department of Pharmacology and Toxicology, School of Medicine, Virginia Commonwealth University, Richmond, VA, United States; ^2^Department of Gastroenterology, Nanhai Hospital, Southern Medical University, Foshan, China; ^3^Department of Internal Medicine, School of Medicine, Virginia Commonwealth University, Richmond, VA, United States; ^4^Department of Gastroenterology, The First Affiliated Hospital of Guangdong Pharmaceutical University, Guangzhou, China

**Keywords:** GLP-1, NLRP3, inflammasome, IL-1β, HMGB1

## Abstract

The glucagon-like peptide-1 (GLP-1) is an insulinotropic hormone secreted by intestinal enteroendocrine L-cells, which plays a crucial role in glucose control, regulation, and protection from different pathological conditions such as diabetes mellitus. The present study sought to test whether GLP-1 release increases gut injury with a high-fat diet (HFD) and whether this GLP-1 release is associated with NLRP3 inflammasome activation. Our results showed that the NLRP3 inflammasome is activated in the intestinal tissue of wild-type mice on a HFD, accompanied by GLP-1 overexpression. The number of intestinal L-cells and the GLP-1 level in serum are increased in WT mice with HFD. However, in the Asc^–/–^ and Nlrp3^–/–^ mice, these HFD-induced intestinal and serum GLP-1 changes were suppressed. Using confocal microscopy, the colocalization of GLP-1 and FLICA that labels activated caspase-1 in intestine was decreased in the Asc^–/–^ and Nlrp3^–/–^ mice compared to WT mice. Mechanistically, the inhibitor of caspase-1 or HMGB1 blocker is used to demonstrate the regulatory action of NRLP3 inflammasome in GLP-1 release. It was found that the level of GLP-1 and its colocalization with IL-1β were reduced by inhibition of the caspase-1 activity, but not altered by blockade of HMGB1 action. Our results suggest that NLRP3 inflammasome activation triggers GLP-1 production from the intestine, which is associated with IL-1β, but not with HMGB1. These findings for the first time provide evidence that the activation of NLRP3 inflammasome in the intestine increases GLP-1 release in mice, which may serve as an adaptive response to intestinal inflammation.

## Introduction

The NLRP3 inflammasome is composed of nod-like receptor (NLR) innate immune cell sensors and responds to toxic signals. It recruits the adapter protein ASC and pro-caspase-1 leading to caspase-1 activation and subsequent secretion of interleukin-1β (IL-1β), IL-18, or high mobility group box-1 protein (HMGB1) (Kamo et al., 2013). The NLRP3 inflammasome activation instigates tissue inflammatory response and thereby plays a significant role in the development of various inflammatory diseases (Yang et al., 2019). It has been reported that the NLRP3 inflammasome senses obesity-associated inducers of caspase-1 activation and thus regulates the magnitude of inflammation and its downstream effect on insulin signaling (Vandanmagsar et al., 2011). Mice exposed to a high-fat diet (HFD) develop acute innate inflammatory responses within hours, reflected by localized IL-1β-dependent accumulation of myeloid cells in the intestine (Progatzky et al., 2014). Modulation of the intestinal microbiota through the regulation of the NLRP3 inflammasome has been shown to be important not only in the regulation of inflammation in diabetic animals but also in glucose homeostasis (Pahwa et al., 2017). During chronic liver injury, inflammasome components were upregulated in liver, but downregulated in gut, which was shown to be associated with microbiota modifications and bacterial translocation (De Minicis et al., 2014). NLRP3 deficiency in the gut is associated with the Western lifestyle diet and can lead to dysbiosis and alteration of the intestinal barrier and bacterial translocation (Pierantonelli et al., 2017). Therefore, there is a complex balance among HFD, gut microbiota, intestinal homeostasis, and NLRP3 function.

The glucagon-like peptide-1 (GLP-1) is an insulinotropic hormone secreted by intestinal enteroendocrine L-cells that regulate insulin and glucagon secretion. GLP-1 is a satiation signal that impacts food intake and weight gain (Duca et al., 2013). GLP-1-based therapies and in particular GLP-1 receptor agonists have proven to be effective in lowering blood glucose and decreasing weight in type 2 diabetes mellitus and obesity patients (Cai et al., 2017). However, the treatment may not be effective due to gut microbiota dysbiosis (Grasset et al., 2017). Such GLP-1 resistance was demonstrated to be mediated through an enteric nitric oxide-dependent and gut–brain axis mechanism. In animal studies, the plasma GLP-1 concentrations were three to four times higher in HFD-fed mice compared to mice fed a normal diet. A GLP-1 analog has been shown to alleviate HFD-induced hepatic steatosis by inhibition of the NLRP3 inflammasome activation (Zhu et al., 2018). To our knowledge, the relationship between NLRP3 inflammasome and GLP-1 release remains unclear. The aim of the present study was to provide an in-depth evaluation of the relationship between the NLRP3 inflammasome activation and GLP-1 released from L-cells during the HFD and to explore the potential action of NLRP3 inflammasome activation on GLP-1 release.

## Materials and Methods

### Animals

In the first series of experiments, C57BL/6J mice (WT), ASC^–/–^ mice, and Nlrp3^–/–^ mice (8 weeks of age, male or female) were fed the normal diets (ND) or HFDs (#D12492, Research Diets, NJ, United States) for 8 weeks. In the second series of experiments, we treated WT mice (8 weeks of age, male or female) with caspase-1 inhibitor (WEHD), HMGB1 inhibitor (GLY), or vehicle used for 2 weeks, started 3 days before they were on a ND or a HFD. This series of experiments was conducted to investigate which products of NLRP3 inflammasome activation is involved in the action on GLP-1 release. All mice were randomly distributed to different experimental groups. At the end of the experimental period, blood samples were collected, and the mice were sacrificed for the harvest of distal intestine tissues, which were used for immunofluorescence staining and biochemical analysis. All protocols were approved by the Institutional Animal Care and Use Committee of the Virginia Commonwealth University.

### Confocal Microscopic Analysis

For confocal analysis of inflammasome molecule colocalization or aggregation, the intestine tissue slides were first fixed in 4% paraformaldehyde in phosphate-buffer saline (PFA/PBS) for 15 min. After being permeabilized with 0.1% Triton X-100/PBS and rinsed with PBS, the slides were incubated overnight at 4°C with anti-NLRP3 (1:200, Abcam, MA, United States) and anti-ASC (1:50, Enzo, PA, United States) or anti-caspase-1 (1:100, Abcam). After washing, these slides were incubated with primary antibodies and were then incubated with Alexa-488- or Alexa-555-labeled secondary antibodies for 1 h at room temperature. The slides were mounted and subjected to examinations using a confocal laser scanning microscope (Fluoview FV1000, Olympus, Japan) with photos being taken, and the colocalization of NLRP3 with ASC or caspase-1 was analyzed by the Image Pro Plus 6.0 software (Media Cybernetics, Bethesda, MD, United States). The summarized data of molecular colocalization efficiency were expressed as correlation coefficient as described previously (Boini et al., 2014), which indicated the formation of NLRP3 inflammasome in the intestine.

### FLICA Staining

To assess the NLRP3 inflammasome activation in the intestine, the tissue slides were processed and labeled with FAM-YVAD-fmk caspase-1 FLICA^TM^ kit (Immunochemistry, Bloomington, IN, United States) according to the manufacturer’s guidelines to detect activated caspase-1. Stained slides were visualized by confocal microscopy for active caspase-1 oligomerization, which was colocalized with cell markers GLP-1 (with antibody staining at 1:100) and in some slides with IL-1β.

### Immunohistochemistry

Intestine tissues were fixed in 4% (v/v) PFA in PBS and embedded with paraffin, which was then sliced into tissue sections (4 μM) and mounted on glass slides. These tissue slides were stained with anti-IL-1β antibody (1:200, R&D Systems, MN, United States), anti-HMGB1 antibody (1:200, Abcam, MA, United States), or anti-GLP-1 (1:200, Abcam, MA, United States) overnight at 4°C after a 20-min wash with 3% H_2_O_2_ and 30-min blocking with 10% serum and then probed with anti-goat Ig-G second antibody labeled with HRP according to the protocols described previously (Xia et al., 2014). Negative controls were prepared without the primary antibodies. The area percentage of the positive staining was calculated in Image Pro Plus 6.0 software. These Immunohistochemistry (IHC) analyses aim at assessing both NLRP3 inflammasome activation and GLP-1 production.

### Western Blot Analysis

Proteins from cell lysates were denatured with SDS buffer and boiled for 5 min. Samples were run on an SDS-PAGE gel, transferred onto polyvinylidene difluoride (PVDF) membrane, and blocked with 5% milk. Then, the membranes were probed with the following primary antibodies overnight at 4°C: mouse anti-GLP-1 (1:1000, Abcam, MA, United States) and rabbit anti-β-actin (1:1000, Santa Cruz, CA, United States). They were incubated with goat anti-rabbit-HRP IgG (1:10,000, Santa Cruz, CA, United States) and goat anti-mouse-HRP IgG (1:10000, Santa Cruz, CA, United States) for 1 h at room temperature. Immunoreactive bands were detected by chemiluminescence techniques after washing three times and then visualized on Kodak Omat X-ray film. The intensity of the specific bands was calculated using ImageJ software version 1.44p. GLP-1 alterations reflect cellular production of GLP-1 in the intestine.

### Statistics

Data were presented as means ± standard error. The significant differences between and within multiple groups were examined using one-way or two-way ANOVA, followed by Duncan’s multiple-range test. *p* < 0.05 was considered statistically significant.

## Results

### IL-1β and HMGB1 Increased in WT Mice With HFD and Were Attenuated in the Intestine of Asc^–/–^ and Nlrp3^–/–^ Mice

**FIGURE 1:**
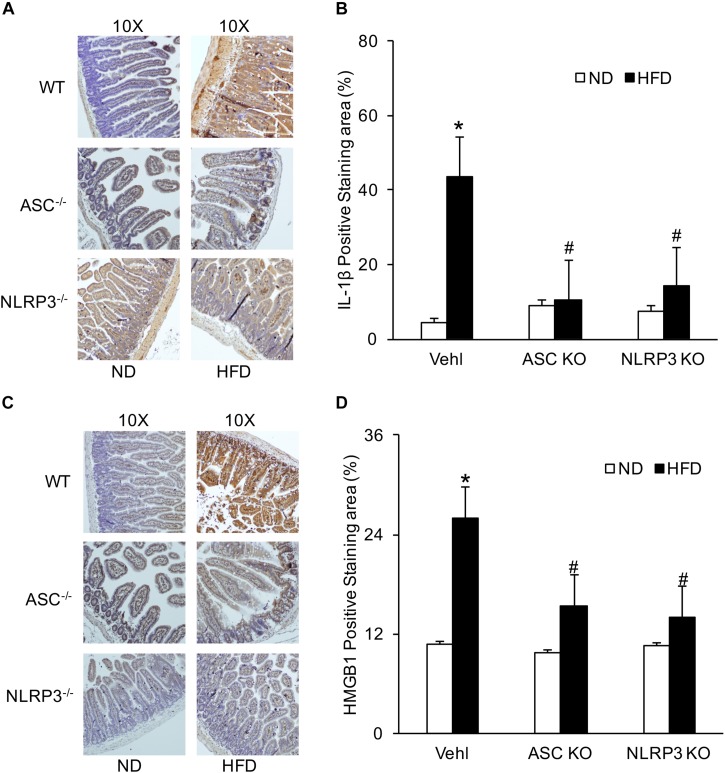
HFD-induced increases in IL-1β and HMGB1 production in intestine of WT, Asc- and Nlrp3-deficient mice. **(A,C)** Representative immunohistochemical images show positive staining of IL-1 β and HMGB1 in intestine of Asc- or Nlrp3-deficient mice and WT mice w/wo HFD. **(B,D)** Summarized data depicting significant increase in positive staining area of IL-1 β and HMGB1 in HFD-WT group, but decrease in Asc- or Nlrp3-deficient mice (*n* = 4 mice per group). **^∗^***p <* 0.05 vs. ND-WT group; **^#^***p <* 0.05 vs. HFD-WT group.

Previous studies have shown that the formation and activation of NLRP3 inflammasome led to the secretion of IL-1β and increase in HMGB1 (Chen et al., 2018). We further confirmed the expression of NLRP3 inflammasome in intestine cells induced by a HFD. By IHC, positive staining of IL-1β and HMGB1 were increased in the intestine of the HFD-treated WT mice group, compared to the ND-treated WT mice group. However, staining of both IL-1β and HMGB1 was decreased in the intestine of Asc^–/–^ and Nlrp3^–/–^ mice fed with a HFD (Figure 1). These results suggested that the NLRP3 inflammasome in the intestine was activated in response to a HFD.

### The Activated Caspase-1 Colocated With GLP-1 in the Intestine of WT Mice With HFD and Was Attenuated in Asc^–/–^ and Nlrp3^–/–^ Mice

**FIGURE 2:**
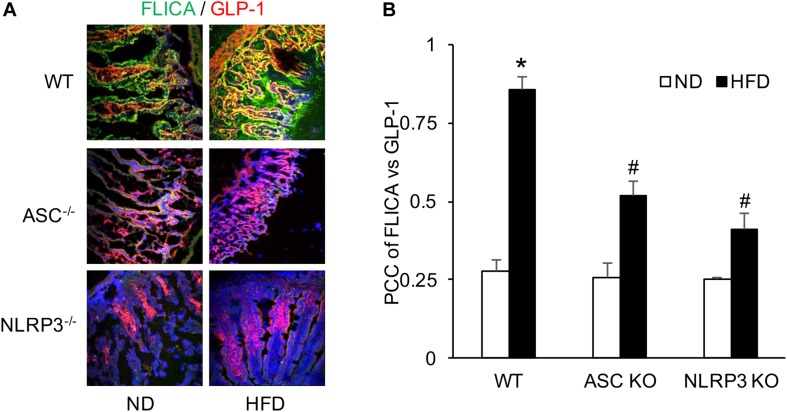
HFD-induced caspase-1 activation measured by FLICA and GLP-1 increases in the intestine of WT, Asc- and Nlrp3-deficient mice. **(A)** Representative images showing active caspase-1 (FLICA) co-localization with GLP-1, the marker of L-cell, which staining in the intestine of Asc- or Nlrp3-deficient mice or WT mice w/wo HFD. **(B)** Correlation coefficient (PCC) showing a statistically significant increase in co-localization of FLICA with GLP-1 in HFD-WT group, but decrease both in Asc- and Nlrp3-deficient mice groups (*n* = 4 mice per group). **^∗^***p <* 0.05 vs. ND-WT group; **#***p <* 0.05 vs. HFD-WT group.

FLICA was performed to detect activated caspase-1 in L-cells of the intestine. It was found that colocalization of activated caspase-1 and GLP-1 were significantly increased in WT mice when compared to mice with ND. However, this HFD-induced increase in colocalization of FLICA and GLP-1 was not observed in both Asc^–/–^ and Nlrp3^–/–^ mice groups. It seems that the HFD induces NLRP3 inflammasome formation and activation in WT mice, which can be blocked by deletion of the gene of Asc or Nlrp3 in mice (Figure 2).

### Increased Number of Intestinal GLP-1-Positive L-Cells During HFD Was Reduced in Asc^–/–^ and Nlrp3^–/–^ Mice With HFD

**FIGURE 3:**
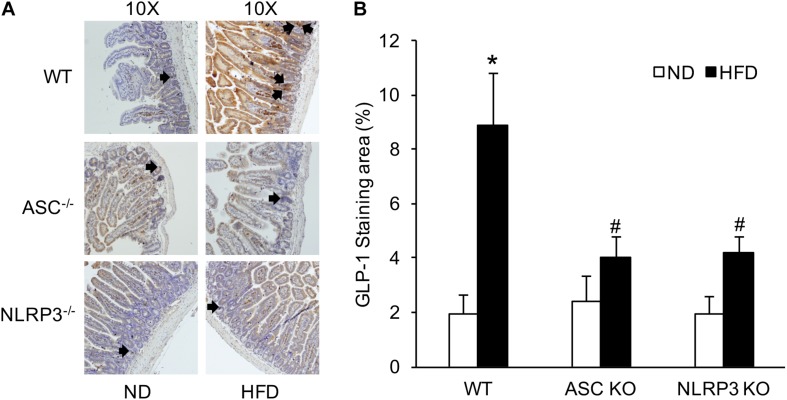
The numbers of intestinal GLP-1 positive L-cells in WT, Asc- and Nlrp3-deficient mice on the HFD. **(A)** Representative immunohistochemical images show the number of intestinal L-cells in Asc- or Nlrp3-deficient mice or WT mice w/wo HFD, the black arrows are the L-cells stained by GLP-1. **(B)** Summarized data depicting significant increase in GLP-1 level in the intestine of WT mice on the HFD, but decrease in Asc-or Nlrp3-deficient mice (*n* = 4 mice per group). ***^∗^****p <* 0.05 vs. ND-WT group; **#***p <* 0.05 vs. HFD-WT group.

L-cells are primarily located in the distal intestine. GLP-1 is a marker of L-cells to indicate the number of L-cells stained by IHC. The number of intestinal GLP-1-positive L-cells was significantly elevated in HFD-treated WT mice compared with ND-treated WT mice. However, the increased number of GLP-1-positive L-cells induced by HFD was downregulated in Asc^–/–^ or Nlrp3^–/–^ mice compared to WT mice with HFD (Figure 3). These results were semi-quantitated by measuring GLP-1-stained areas in the intestinal tissue. It is supposed that deletion of a major gene encoding the NLRP3 inflammasome component attenuates HFD-induced increases in the number of GLP-1-positive L-cells.

### HFD-Induced Increases in the Level of Serum GLP-1 Disappeared in Asc^–/–^ and Nlrp3^–/–^ Mice

**FIGURE 4:**
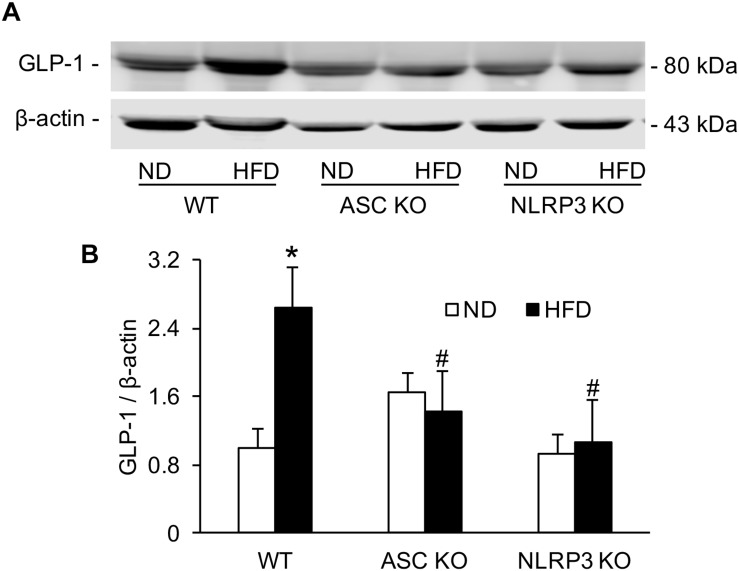
The level of serum GLP-1 in WT, Asc- and Nlrp3-deficient mice on the HFD. **(A)** Representative Western blots gel documents showing GLP-1 in serum of Asc or Nlrp3-deficient mice and WT mice w/wo HFD. **(B)** Densitometric quantitation of immunoreactive bands showing GLP-1 has a statistically significant increase in HFD-WT group, but decrease in Asc or Nlrp3-deficient mice group (*n* = 4 mice per group). *^∗^p <* 0.05 versus ND-WT group; #*p <* 0.05 versus HFD-WT group.

GLP-1 level in serum was analyzed by Western blot. Densitometric quantitation of immunoreactive bands showed that GLP-1 levels were significantly increased in the HFD-WT mice group compared to the ND-WT mice group. However, in Asc^–/–^ and Nlrp3^–/–^ mice, this HFD-induced GLP-1 increase was not observed (Figure 4).

### HFD-Induced NLRP3 Inflammasome Formation in the Intestine

**FIGURE 5:**
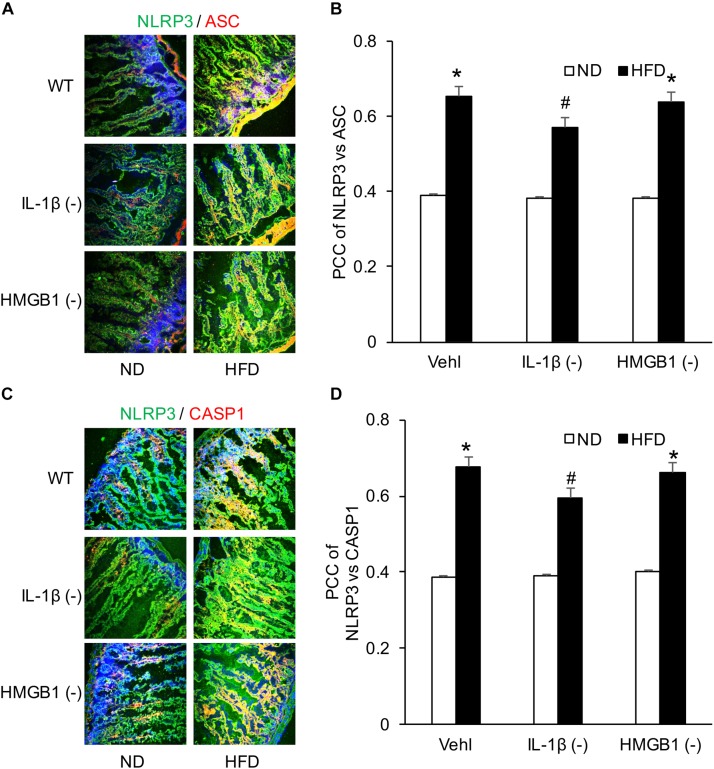
HFD-induced NLRP3 inflammasomes formation in the intestine before and after treatment of caspase-1 inhibitor (WEHD) and HMGB1 blocker (GLY). **(A,C)** Representative fluorescence confocal microscopic images showing the co-localization of NLRP3 and ASC or caspase-1 in intestine when pretreat with caspase-1 inhibitor (WEHD), and HMGB1 inhibitor (GLY), and Vehl. **(B,D)** PCC showing a statistically significant increase in co-localization of NLRP3 and ASC or caspase-1 in HFD-Vehl and HFD-GLY groups, but decrease in HFD-WEHD group (*n* = 4 mice per group). **^∗^***p <* 0.05 vs. ND-Vehl group; **#***p <* 0.05 vs. HFD-Vehl group.

NLRP3 inflammasome activation led to the release of IL-1β and HMGB1, which may increase the release of GLP-1 from L-cells. To explore this mechanism, we performed another series of experiments to test whether GLP-1 release is associated with NLRP3 inflammasome activation and which inflammasome products such as IL-1β or HMGB1 contribute to GLP-1 release. It was demonstrated that HFD induced NLRP3 inflammasome formation as shown by colocalization of NLRP3 with ASC or caspase-1 in WT mice, which were not significantly blocked by caspase1 inhibitor (WEHD) or HMGB1 inhibitor (GLY) (Figure 5).

### IL-1β and HMGB1 Increased by HFD in Intestine Were Significantly Attenuated by WEHD or GLY

**FIGURE 6:**
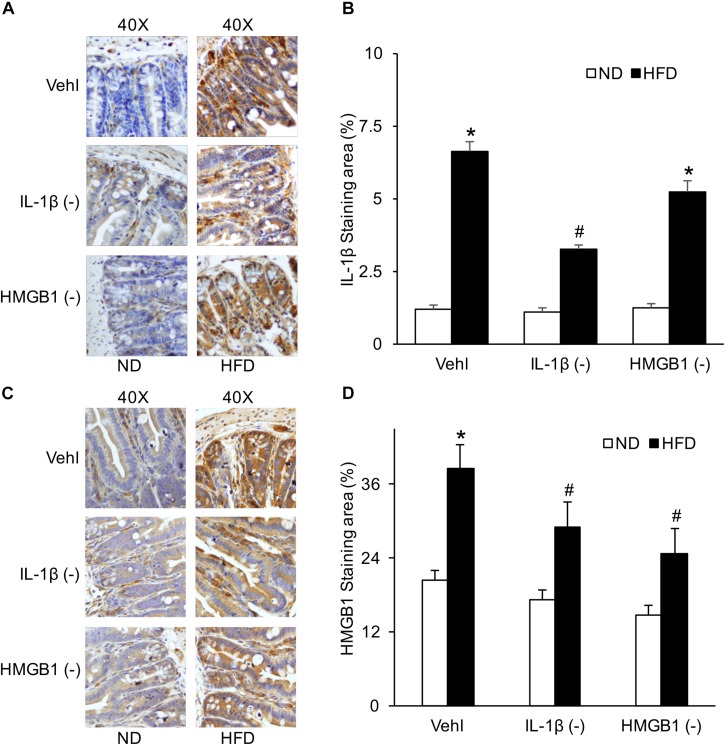
IL-1β and HMGB1 levels in the intestine before and after treatment of caspase-1 inhibitor (WEHD) and HMGB1 blocker (GLY). **(A)** Representative immunohistochemical images show positive staining of IL-1β in intestine of WT mice w/wo HFD when pretreated with WEHD, and GLY, and Vehl. **(B)** Summarized data of IL-1β positive area measurements depicting significant increase both in HFD-Vehl and HFD-GLY groups, but decrease in HFD-WEHD group. **(C)** Representative immunohistochemical images show positive staining of HMGB1 in intestine of WT mice w/wo HFD when pretreated with WEHD, and GLY, and Vehl. **(D)** Summarized data depicting significant increase in positive staining area of HMGB1 in HFD-Vehl group, but decrease both in HFD-WEHD and HFD-GLY groups (*n* = 4 mice per group). **^∗^***p <* 0.05 vs. ND-Vehl group; **^#^***p <* 0.05 vs. HFD-Vehl group.

By detecting the product of the NLRP3 inflammasome, IL-1β, and HMGB1, HFD-induced NLRP3 inflammasome activation in the intestine was found to be blocked by caspase-1 inhibition with WEHD or HMGB1 binding blocker, respectively. Both WEHD and GLY attenuated HFD-induced increases in the HMGB1 level (Figures 6C,D), but only WEHD blocked HFD-induced IL-1β production (Figures 6A,B and Supplementary Figure S1). It was confirmed that IL-1β production is blocked by the inhibitor of caspase-1, but not by the HMGB1 inhibitor.

### HFD Increased the Colocalization of IL-1β and GLP-1 in Intestine, but Had No Effect on the Colocalization of HMGB1 and GLP-1

**FIGURE 7:**
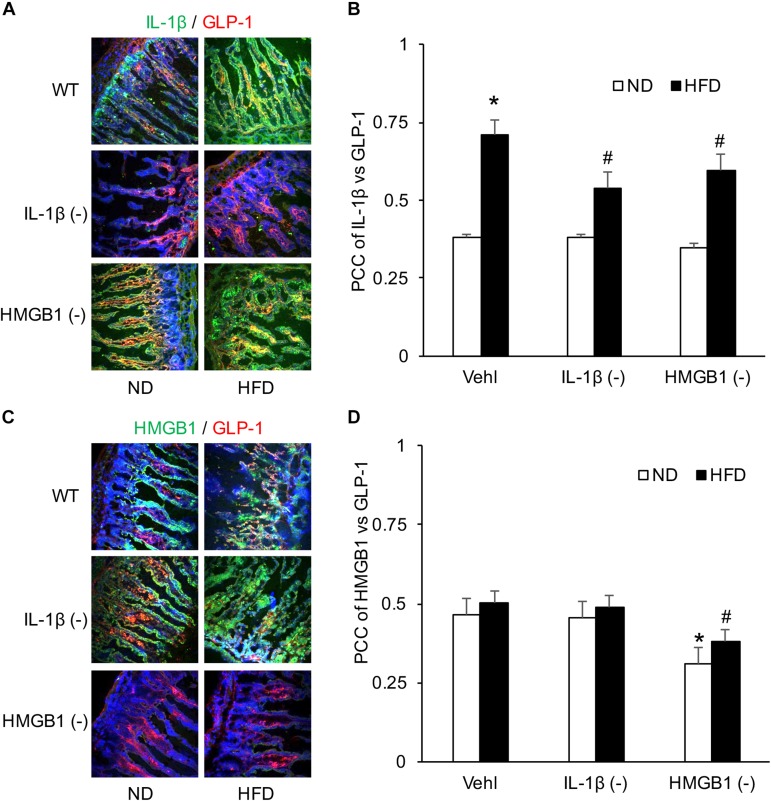
Co-localization of IL-1β and GLP-1 in the intestine during the HFD in mice with or without treatment of caspase-1 inhibitor (WEHD) and HMGB1 blocker (GLY). **(A)** Representative images showing IL-1 β co-localization with GLP-1 in the intestine of WT mice w/wo HFD pretreat with WEHD, and GLY, and Vehl. **(B)** PCC showing a statistically significant increase in co-localization of IL-1β and GLP-1 in HFD-Vehl group, and decrease in HFD-WEHD and HFD-GLY groups. **(C)** Representative images showing HMGB1 co-localization with GLP-1 in the intestine of WT mice w/wo HFD pretreat with WEHD, and GLY, and Vehl. **(D)** PCC showing a statistically significant increase in co-localization of HMGB1 and GLP-1 in HFD-Vehl and HFD-WEHD groups, but decrease when pretreated with GLY (*n* = 4 mice per group). **^∗^***p <* 0.05 vs. ND-Vehl group; **#***p <* 0.05 vs. HFD-Vehl group.

After confirmation of the formation and activation of NLRP3 inflammasome, confocal analysis was used to detect the relationship of IL-1β or HMGB1 to GLP-1. Representative images and summarized data showed that HFD increased colocalization of IL-1β with GLP-1 in the intestine, which was attenuated by treatment of either WEHD or GLY (Figures 7A,B). On the contrary, there was no significant effects of HFD on the colocalization of HMGB1 with GLP-1 despite the fact that GLY could reduce this colocalization in the intestine (Figures 7C,D). These results suggest that HFD-induced GLP-1 increase may be associated with IL-1β production, but not with HMGB1 release during NLRP3 inflammasome activation.

### GLP-1 Expression in the Intestine When Mice Were Treated With WEHD and GLY

**FIGURE 8:**
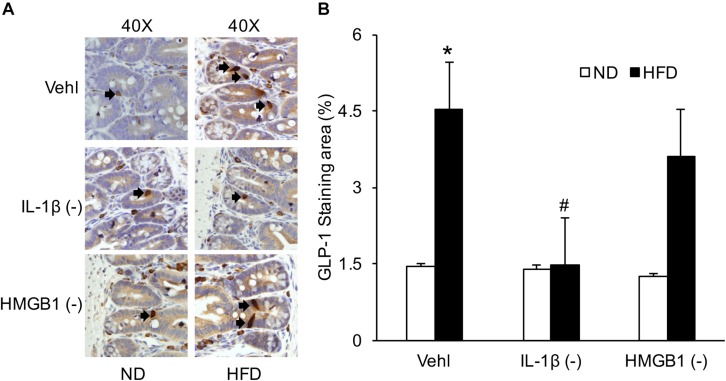
GLP-1 positive L-cells in the intestine during the HFD in mice with or without treatment of caspase-1 inhibitor (WEHD) and HMGB1 blocker (GLY). **(A)** Representative immunohistochemical images show positive staining of GLP-1 in intestine of WT mice w/wo HFD when pretreated with WEHD, and GLY, and Vehl. **(B)** Summarized data depicting significant increase in positive staining area of GLP-1 in HFD-Vehl and HFD-GLY groups, but decrease both in HFD-WEHD group (*n* = 4 mice per group). **^∗^***p <* 0.05 vs. ND-Vehl group; #*p <* 0.05 vs. HFD-Vehl group.

By IHC, we verified the number of L-cells in the intestine to confirm HFD-induced GLP-1 increase, which were associated with IL-1β, but not with HMGB1. As shown in Figure 8, representative images and summarized data showed that HFD increased the positive staining area of GLP-1 or GLP-1-positive cells, which were blocked by treatment of mice with WEHD, but not by GLY. This further confirmed that NLRP3 inflammasome activation during HFD stimulates the release of GLP-1 through its product IL-1β, but not HMGB1.

## Discussion

In the present study, we provide several lines of evidence indicating that the intestinal NLRP3 inflammasome activation with a HFD contributes to GLP-1 release from L-cells. First, the activation of NLRP3 inflammasome in the intestine plays an essential role in gut injury and inflammation in mice exposed to the HFD. Also, NLRP3 inflammasome activation stimulates GLP-1 production and release from the L-cells of the intestine. Finally, IL-1β, but not HMGB1, is an important NLPR3 inflammasome product that stimulates GLP-1 release. Therefore, HFD-induced NLRP3 inflammasome activation in the intestine may lead to GLP-1 production and release via IL-1β in L-cells of the intestine, which may be an important adaptive response to dysbiosis during HFD.

It has been reported that HFD may directly induce inflammasome activation and thereby instigate intestinal inflammation (Progatzky et al., 2014). Moreover, NLR plays a critical role in the mucosal defense in the gut (Elia et al., 2015). Accumulating evidence suggests that the inflammasome is pivotal in shaping epithelial responses at the host–lumen interface, where many inflammasome components are highly expressed by intestinal epithelial cells (Lei-Leston et al., 2017). The present study demonstrated that the NLRP3 inflammasome is activated in intestinal epithelial cells during HFD. By knocking out the Nlrp3 and Asc gene in mice, HFD-induced formation and activation of NLRP3 inflammasome in the intestine were suppressed. Consistently, a caspase-1 inhibitor, WEHD, also showed the same effect. There is evidence that failure to activate the NLRP3 inflammasome can lead to gut–liver axis derangement, gut dysbiosis, and a worsened phenotype in a mouse model of non-alcoholic fatty liver disease (NAFLD) (Henao-Mejia et al., 2012; Pierantonelli et al., 2017). Hence, the endocrine system and inflammation in the gut can promote chronic inflammatory diseases of the liver. However, the precise mechanism of this intestinal inflammasome activation and associated pathological or adaptive response remains unclear.

The present study surprisingly found that intestinal NLRP3 inflammasome activation during the HFD stimulates GLP-1 production and release. GLP-1 is a 30-amino-acid-long peptide hormone derived from the tissue-specific posttranslational processing of a pro-glucagon peptide. It is packaged in secretory granules and secreted into the hepatic portal system by intestinal L-cells located primarily in the distal ileum and colon, but also found in the jejunum and duodenum (Hansen et al., 2013). L-cells and GLP-1 functions correlate with the effects of incretin-based therapies on metabolic and inflammatory signaling (Zietek and Rath, 2016). GLP-1 serves a role in inflammation and may inhibit lipopolysaccharide-induced cytokine secretion in various types of cells (Sun et al., 2017). GLP-1 plasma levels correlate with markers of inflammation and disease severity, and it provides a link between the endocrine system and the gut with strong relevance for metabolic regulation in the context of inflammation (Kahles et al., 2014). Fatty acid–induced NLRP3-ASC inflammasome activation interferes with insulin signaling (Wen et al., 2011). We demonstrated that the number of L-cells and GLP-1 levels in serum were reduced in the Nlrp3^–/–^ and Asc^–/–^ mice treated with HFD compared with WT mice, suggesting that HFD-induced enhancement of liver injury or other metabolic damages in the Nlrp3^–/–^ and Asc^–/–^ mice may be due to the loss of GLP-1-mediated protective mechanisms. It seems that the NLRP3 inflammasome activation in the intestine exerts a critical defending action during HFD, which protects the liver or other organs from metabolic injury through increased production or release of GLP-1.

We further addressed which main products from NLRP3 inflammasome activation change the formation and release of GLP-1 from the intestinal L-cells. It was found that IL-1β, rather than HMGB1, mainly contributes to the GLP-1 production and release from these L-cells because the inhibition of the IL-1β-producing enzyme, caspase-1, blocked increases in GLP-1-positive L-cells in the intestine and serum GLP-1 level. Although there are no studies that elucidate the role of NLRP3 inflammasome activation in GLP-1 production and release, previous studies have shown that GLP-1 secretion was indeed altered by a variety of inflammatory stimuli, including IL-1β, endotoxin, and IL-6 (Kahles et al., 2014). Based on these results, we assumed that the intestinal innate endocrine response to potential inflammatory changes in gut may stimulate GLP-1 production and release, which may exert a protective role in the development of metabolic disorders. Without innate endocrine response of inflammation, there will be a risk for development of liver injury and metabolic disturbances. However, some showed increases in GLP-1 production and release by HFD, but others demonstrated reduction of its formation and secretion by HFD (Wang et al., 2015). These controversial reports may be related to the status of gut microbiota and NRLP3 inflammasome in different experimental animals that were exposed to HFD.

## Conclusion

The present study demonstrated that increased NLRP3 inflammasome formation and activation may be an important host defense response to gut dysbiosis induced by HFD, which regulates the quantity of GLP-1-positive L-cells and the secretion of GLP-1 through IL-1β. The deficiency of such NLRP3 inflammasome-mediated gut defense response may block GLP-1 formation and release, thereby linking gut dysbiosis and metabolic disorders.

## Data Availability Statement

The raw data supporting the conclusions of this manuscript will be made available by the authors, without undue reservation, to any qualified researcher.

## Ethics Statement

The animal study was reviewed and approved by Ethics Committee of VCU.

## Author Contributions

YC, JK, and P-LL contributed conception and design of the study. YC, JH, and OB organized the database. CY and XY performed the statistical analysis. CY wrote the first draft of the manuscript. JK, JH, XH, and P-LL wrote the sections of the manuscript. All authors contributed to manuscript revision, and read and approved the submitted version.

## Conflict of Interest

The authors declare that the research was conducted in the absence of any commercial or financial relationships that could be construed as a potential conflict of interest.

## Supplementary Material

FIGURE S1 Summarized data of serum IL-1; detected by ELISA depicting significant increase both in HFD-Vehl and HFD-GLY groups, but decrease in HFD-WEHD group (*n* = 4 mice per group). ^∗^*p* < 0.05 vs. ND-Vehl group; ^#^*p* < 0.05 vs. HFD-Vehl group.
