# Supplementary figures and images for: Suppression of Glucagon-Like Peptide-1 Release by Inhibition of Intestinal NLRP3 Inflammasome Activation in Asc–/– and Nlrp3–/– Mice
Source: Front Physiol. 2019 Oct 1;10:1213. doi: 10.3389/fphys.2019.01213 (PMC6779826; doi:10.3389/fphys.2019.01213)

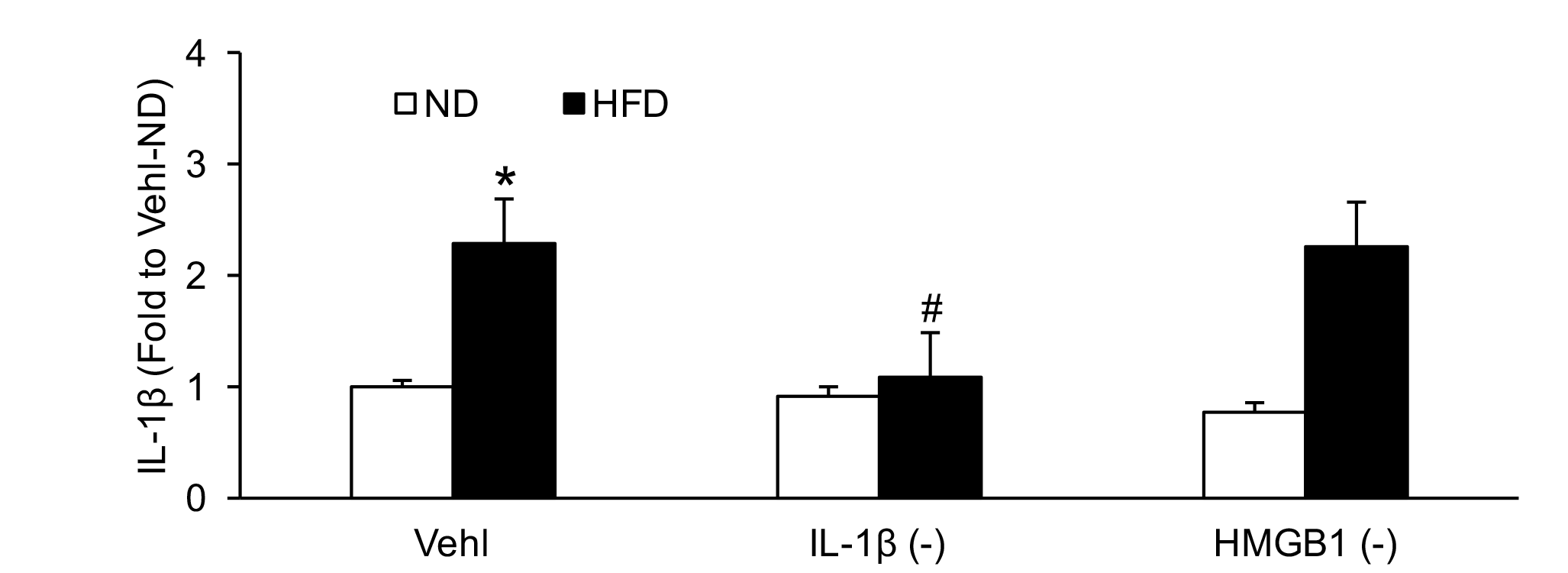

Supplement: FIGURE S1 — Summarized data of serum IL-1; detected by ELISA depicting significant increase both in HFD-Vehl and HFD-GLY groups, but decrease in HFD-WEHD group (n = 4 mice per group). ∗p < 0.05 vs. ND-Vehl group; #p < 0.05 vs. HFD-Vehl group. [file Image_1.TIF]
